# Reviews

**Published:** 1922-11

**Authors:** 


### REVIEWS OF BOOKS.

**THE POETIC MIND.** By FREDERICK C. PRESCOTT. Pp. xxii; 308. (Macmillan Co., New York.)

This is an excellent book. Much as we may dissent from some of the opinions set forth by Prof. Prescott, there is no denying that he has treated an interesting theme in a masterly way. Nothing in literature has been dealt with in such an obfuscating manner as the workings of the poet's mind. So long as the idea of some mysterious "inspiration" holds sway there can be no rational explanation. The Freudian school of thought has certainly done good in so far as it has called attention to the workings of the subconsciousness—or the "unconscious," as it is designated. That the greater part of mental action is subconscious is not, of course, a new conception; but it is a fact which has not been duly appreciated by those who directed their attention to the examination of consciousness.

Prof. Prescott applies these theories to the poetic mind in particular, regarding pure poetry as "a product of a mental operation quite different from that which produces mere verse or ordinary prose"—a quite arbitrary distinction, but one which need not necessarily invalidate his results. He rather disarms criticism, by the way, by stating that he has no special psychological training; and one might be inclined, therefore, to read his book more for amusement than for instruction—and be glad, in a welter of unnecessary volumes on pseudo-psychology, even to be amused! In any case he need not apologise in these days for such a lack—even if his book gave evidence of it.

Two modes of thought are described. The first is associative thought, "consisting of a succession of images, one calling up another freely and spontaneously, by either contiguity or similarity." It is the more primitive type of thinking, and approximates to what occurs in musing and dreaming. The second is voluntary or purposive thought, "which is guided by a distinct purpose or conscious interest, controlling by selection the associations spontaneously offered, to fit the end in view. The will, impelled by interest, fixes the attention, and this results in reasoning." The first is more subjective—it "has a character of withdrawal and inwardness"; while voluntary thought is more objective—"it is closely in touch with sensuous experience, immediately utilising the reports of the senses and controlling them." Associative thought is the mode—generally speaking—which is utilised in the production of poetry; though, "the two kinds of thought run together by a gradation." And it must not be forgotten that "the mind is a vital unity easily misrepresented by sharp distinctions." It is obvious, therefore, that, in order to make use of this method of explanation, it will be necessary to define what is meant by poetry—or, as Prof. Prescott puts it "essential poetry." It might include, however, what has been described as the "Asylum School of Poetry" as well as poetry conforming to more classical rules!

It will be seen that, in both instances, the author does not invoke the extraneous "inspiration" to explain the production of poetry. What some regard as a descent of winged words from the empyrean is in reality an energetic uprush into consciousness from subliminal regions. And there is not a mystery about where the material for the formation of images comes from—"there must be a fund of images to be drawn upon, which the poet must at some time have got from experience." One reason why this accumulated energy issues in the form of poetry is that, in civilised society at least, desires are denied expression directly, and they are forced, therefore, to find gratification in an indirect manner. If they do not succeed in obtaining gratification, great emotional disturbance may result, and this, if high in degree, may show itself as poetic madness.

Prof. Prescott supports his contentions by means of quotations drawn from many sources, and aptly applied. In every way his book gives evidence of a scholarly mind; and from the literary point of view it is fascinating. That it is the final word from the scientific aspect is another matter; and this seems to be implied in the author's deprecation of "scientific research" and "laboratory methods" in literary study, as also in his statement that "psychology must obtain most of its facts ultimately from introspection." How many "facts" have been obtained by the introspective method? And what amount of advance in knowledge of brain function took place before the era of scientific investigation? Only by the correlation of results obtained by *all* methods of research shall we acquire accurate information as to the working of the poetic—or prosaic—mind in health and in disease; and mental hygiene will inevitably benefit from such increase of knowledge. Meantime—the thanks of the reviewer to the author.—H. J. N.

---

**MEDICAL INSURANCE PRACTICE.** By R. W. HARRIS and L. SHOETEN SACK. (The Scientific Press, Ltd.; 7s. 6d. net.)

Doctors are notoriously lax in the care with which they read the various documents that come to them from Government Departments and from Local Government Authorities. This is true of many insurance practitioners even with regard to Regulations and Memoranda which will obviously affect the conditions of their practice and possibly their remuneration. To have, collected in one handy volume, all that it is really important for them to know about the administration, supervision and conduct of the insurance service will be a great boon to most doctors, and certainly a convenience even to those who have already made themselves familiar with the terms and conditions of their service and with the powers and the practical working of the various committees concerned therewith. This is what Messrs. Harris and Shoeten Sack have done for them in their little book on Medical Insurance Practice. The book is admirably arranged, clearly printed, well indexed, and convenient to handle both as to form and binding. The system of marginal references is excellent, and the interleaving of that portion which sets out the official "Terms of Service" will enable the careful doctor to note those minor, though important, changes which may from time to time be made in this section of the Medical Benefit Regulations.

The book, of course, has no official status, but this is no loss, since the intimate experience of the authors with that Department of the Ministry of Health concerned with their subject matter enables them to speak with authority, and yet to re-arrange and simplify the official material in a way which smooths the difficulties which some insurance doctors seem to find in mastering it. A careful reading of the volume fails to disclose any important omissions or any real inaccuracy. A rare ambiguity or apparent contradiction appears to be due to a lack of precision in the original documents or procedure rather than to any fault in the exposition.

Every insurance practitioner ought to possess this book and to use it, but it should be of interest and use to many others. The newly-qualified student who is about to choose his branch of medical practice, and those who may be looked to for help in making the choice, will find it an advantage to consult such a succinct review of the position and requirements of the insurance service. Many of those, too, both medical and lay, who take part in the administration of the service may be glad of a similar advantage. The general view which the authors enable us to take reveals not merely the perhaps too complicated character of some of the arrangements made, but the real care which is taken to secure for insured persons adequate medical attention within that wide field known as "general practitioner treatment." The important and interesting extracts which the authors incidentally give from documents published by the Insurance Acts Committee of the British Medical Association indicate that those who speak for the doctors take no narrow view of their sphere and responsibility, and have a zealous care for the honour of the profession, and the good name of the service. We can unreservedly congratulate both authors and publishers on having produced a volume which worthily supplies a real need.—B.

---

**A TEXT-BOOK OF BACTERIOLOGY.** By HANS ZINSSER, M.D. Fifth edition. Pp. 1,194. (D. Appleton and Co.; 35s. net.)

This book has been completely revised and rewritten since the fourth edition was published four years ago. It is no dry description of morphology and culture-habits of bacteria, but a thoroughly up-to-date description of pathogenic organisms and protozoa, with adequate and interesting notes upon the clinical and immunological factors which they involve. To the bacteriologist whose thoughts are not confined within the walls of his laboratory this book should make a strong appeal, and it is also a valuable work of reference for the clinician, whose knowledge of pure bacteriology must, of necessity, be limited. The information offered is reliable and up to date; it is evident that great care has been taken in winnowing from the mass of publications which continually accumulates the chaff of the useless or the doubtful. Views which are entitled to the benefit of the doubt receive sufficient mention; those which have not this claim are wisely ignored.

In dealing with influenza the author states of Pfeiffer's Bacillus, "We feel confident that if the disease is a bacterial disease at all, no other bacteria need be seriously considered." Later, after a judicial discussion of the possible part played by a filterable virus, he refers to the "considerable amount of evidence which should make one conservative in definitely claiming etiological relationship for the influenza bacillus." This is a good example of the impartial discussion of conflicting hypotheses which marks the tone of the whole book. It is difficult to single out any sections for special comment, but, in view of their wide application, the discussion of the Schick reaction, the prophylactic immunisation against diphtheria by combined toxin and anti-toxin injections, may be mentioned. The book is well got up, the print is clear and the style good. We recommend it strongly.—C. E. S.

---

**ANATOMY AND PHYSIOLOGY FOR JUNIOR NURSES.** By FÉLICIE NORTON, Author of "Clinical Notes for Probationers," etc. (The Scientific Press, Ltd.; 2s. 9d. post free.)

In this little book much has been done to simplify what is difficult and complex; it is, indeed, so simplified as at times to omit what should be included for a proper understanding of the subject, for the sake of brevity. However, the essentials are there, and the author has set them forth in such a manner as to smooth considerably the rough path for beginners besides whetting the appetite for fuller knowledge. Written by one who has been a sister-tutor herself and based on lectures delivered by her to her class of hospital pupils, the book should be useful to others less experienced in the art of teaching. Miss Norton has a full knowledge of the difficulties that confront the young nurse with no previous knowledge of anatomy and physiology, and a clear method of dealing with them. A number of simple diagrams scattered throughout the book will be found helpful in elucidating knotty points. The chapters should be amplified by the teacher, and the book studied alongside with the lectures, or together with a slightly more advanced text book on the subject. Its low price will recommend it to intending probationers.

E. M. F.

---

**NOTES ON GYNÆCOLOGICAL NURSING.** By FÉLICIE NORTON, Author of "The Midwife's Companion," etc. (The Scientific Press, Ltd.; 1s. 4½d. post free.)

This small volume abounds in sound practical information on the subject of which it treats, and is written by a well-known expert. Miss Norton gives in few words the explanation of all technical gynæcological terms so often but vaguely comprehended by the student. She describes tersely the anatomy and physiology of the female generative organs, certain diseased conditions and their special treatment, the meaning and importance of symptoms, and gives detailed descriptions of all the usual gynæcological and obstetric operations, with instruments required. The chapters are amplified from articles which appeared originally in the "Nursing

Times," and in their present form of a compact little book, take honourable place in the "S. P. Pocket Guide Series." Miss Norton's writings are marked by clearness and simplicity, and show signs of a wide experience and careful observation. Her remarks on the menopause in the chapter on disorders of menstruation are sensible and vigorous. To many women, she says, the dread of this period is as a threatening shadow hanging over their lives, but it need not be so. The menopause is but a natural phenomenon, and its discomforts merely transient. Change of life is, however, not normally accompanied by "floodings," as is so commonly and erroneously supposed. Loss of blood between the periods may be caused by a fibroid or cancerous growth. By persuading patients to report any such hæmorrhage to a doctor, nurses may be instrumental in saving many lives. No nurse, gynæcological or otherwise, but will find her sense of responsibility quickened and deepened by the careful perusal of this little book.—E. M. F.

---

**THE TREATMENT OF INJURIES OF THE PERIPHERAL SPINAL NERVES.** By Sir Harold Stiles and Dr. M. F. Forrester-Brown. Pp. 180; with over 60 illustrations. (Oxford Medical Publications, Henry Frowde and Hodder and Stoughton; 15s. net.)

This is one of the best of the *post-bellum* monographs. Not only have we the surety of good work and sound lines in the name of Stiles, but we also have in the junior author accuracy of detail, with very clear and artistic illustrations of the actual cases, taken at the time of operation. There is no padding in the book, clear issues are stated, and the best way to overcome every conceivable difficulty. Surgeons see few of these nerve injuries in civil practice; but, with this book before him, any general surgeon could successfully deal with any lesion. More than a mere word of praise is due for the excellence of the coloured sketches, and the book shows very clearly the advantages of these over photographs, however well the latter may be taken and reproduced, and reproduction is not easy in books. The few errors in the printing are not sufficient to detract from the value of the work to the experienced surgeon. We have no hesitation in stating that no hospital surgeon can afford to be without this work, as a bad result may, in these cases, land any operator in a law-suit. It is too little to express our thanks to the writers; they have put the profession under a deep debt of gratitude to both of them.—A. D.

---

**DENTAL RADIOLOGY.** By C. Kempster, M.R.C.S., L.R.C.P. (The Scientific Press, Ltd.; 10s. 6d. net.)

While there can be no doubt that concealed dental sepsis is a cause of much ill-health, there is a widespread feeling that some of the dental executions which are so common nowadays are carried out without fair trial of the accused, and without convincing evidence of guilt. Unfortunately it is not always possible to say upon ordinary examination whether disease is present at the root of a tooth; an apparently normal tooth may indeed be a whited sepulchre. Dental radiography affords the information needed, it brings home guilt to the guilty and acquits the innocent. This clearly written and well illustrated handbook includes in its scope descriptions of the necessary apparatus, the methods adopted in taking dental radiograms, and the interpretation of the pictures obtained. It should be of value not only to the dental surgeon, but also to the general physician or surgeon to whom the patient looks for advice as to "whether the tooth should come out."

C. E. S.

---

**INFLUENZA.** Essays by Several Authors. Edited by F. G. Crookshank, M.D., F.R.C.P. (Wm. Heinemann, Medical Books, Ltd.; 30s net.)

This is no ordinary book. Dr. Crookshank has set himself the task of presenting in a single volume a complete survey of influenza in its historical, epidemiological, bacteriological and clinical aspects. He has obtained the assistance of experts in the different fields, but their contributions dovetail in unusually well. Dr. Crookshank holds that influenza includes other various conditions described as botulism, encephalitis lethargica, polyneuritis, purulent bronchitis, even perhaps, trench fever. He shows that outbreaks of unfamiliar diseases have always occurred in relation to influenza-periods and that these have usually been considered to be manifestations of some kind of specific new disease. This thesis is elaborated in a striking article by Hamer, who also joins with the editor in reviving the old idea of an epidemic constitution. The claims for Pfeiffer's bacillus as the cause of influenza receive their death-blow in the 160 pages contributed by Donaldson. The suggestion of a filter-passing virus is likewise considered judicially and, in turn, rejected as untenable.—C. E. S.

---

**PNEUMONIA.** By Frederick Taylor Lord. (Harvard Health Talks Series: Cambridge Harvard University Press; 4s. 6d. net.)

This little manual is written for the qualified and the unqualified. A clear statement is given of the clinical value and applications of the grouping of pneumococci into four types. It is pointed out that, while type I is present in 30 per cent. of cases of lobar pneumonia, it is a relatively rare passenger in the normal mouth; that while approximately 33 per cent. of cases of type I are fatal, it is possible by administration of type I serum to reduce this mortality to 10 per cent. With regard to the other types of pneumococcal infection, no benefit is to be expected from serum treatment. The dangers of serum treatment are clearly pointed out. Sound lines of hygiene are advocated. The lecture on which this booklet was based must have been a great success; in its permanent form it is of value as a considered statement of facts with reference to the treatment of pneumonia upon scientific lines.—C. E. S.

---

"Adventures of a South African Nursing Sister." By F. M. Ayliff. (Arthur H. Stockwell, 5s. net.) Nurses will find much to interest and amuse them in this unpretentious little book, with its many sketches of hospital and private work in South Africa.

